# Cellular Distribution of Aquaporin 3, 7 and 9 in the Male Reproductive System: A Lesson from Bovine Study (*Bos taurus*)

**DOI:** 10.3390/ijms25031567

**Published:** 2024-01-26

**Authors:** Patrycja Oberska, Marta Grabowska, Marta Marynowska, Maciej Murawski, Dariusz Gączarzewicz, Andrzej Syczewski, Katarzyna Michałek

**Affiliations:** 1 Department of Physiology, Cytobiology and Proteomics, West Pomeranian University of Technology in Szczecin, Klemensa Janickiego 29, 71-270 Szczecin, Poland; patrycja.oberska@zut.edu.pl (P.O.); marta.marynowska@zut.edu.pl (M.M.); 2 Department of Histology and Developmental Biology, Pomeranian Medical University, Żołnierska 48, 71-210 Szczecin, Poland; marta.grabowska@pum.edu.pl; 3 Department of Nutrition, Animal Biotechnology and Fisheries, University of Agriculture in Krakow, 24/28 Mickiewicza Avenue, 30-059 Cracow, Poland; rzmmuraw@cyf-kr.edu.pl; 4 Department of Animal Reproduction, Biotechnology and Environmental Hygiene, West Pomeranian University of Technology in Szczecin, Klemensa Janickiego 29, 71-270 Szczecin, Poland; dariusz.gaczarzewicz@zut.edu.pl; 5 Genetic and Animal Husbandry, Tomasza 40, 72-006 Szczecin, Poland; biuro@bydlo-as.pl

**Keywords:** water channel, morphology, GATA-4, male reproductive potential, testis, epididymis, vas deferens

## Abstract

The increasing incidence of male infertility in humans and animals creates the need to search for new factors that significantly affect the course of reproductive processes. Therefore, the aim of this study was to determine the temporospatial expression of aquaglyceroporins (AQP3, AQP7 and AQP9) in the bovine (*Bos taurus*) reproductive system using immunohistochemistry and Western blotting. The study also included morphological analysis and identification of GATA-4. In brief, in immature individuals, AQP3 and AQP7 were found in gonocytes. In reproductive bulls, AQP3 was observed in spermatocytes and spermatogonia, while AQP7 was visible in all germ cells and the Sertoli cells. AQP7 and AQP9 were detected in the Leydig cells. Along the entire epididymis of reproductive bulls, aquaglyceroporins were visible, among others, in basal cells (AQP3 and AQP7), in epididymal sperm (AQP7) and in the stereocilia of the principal cells (AQP9). In males of all ages, aquaglyceroporins were identified in the principal and basal cells of the vas deferens. An increase in the expression of AQP3 in the testis and cauda epididymis and a decrease in the abundance of AQP7 in the vas deferens with age were found. In conclusion, age-related changes in the expression and/or distribution patterns of AQP3, AQP7 and AQP9 indicate the involvement of these proteins in the normal development and course of male reproductive processes in cattle.

## 1. Introduction

More than 20 years have passed since 2001, when Calamita and co-workers were among the first to draw attention to the fact that multiple aquaporins (AQPs) in the male reproductive system could play an important role in the normal course of reproductive processes [1]. Although over the years a number of interesting studies have been published, suggesting, among others, the involvement of AQPs in testicular development, spermatogenesis and sperm physiology, the significance of these proteins within male reproductive organs has still not been fully elucidated and proven [2]. Currently, in the face of the growing global increase in male infertility, both in humans and animals, and the necessity to explore new factors significantly affecting male reproductive potential, AQPs have become the focus of interest for many researchers.

It is widely known that AQPs belong to the family of small, integral proteins that facilitate the bidirectional transport of water and a variety of small, uncharged solutes across cell membranes. In modern placental mammals, 13 AQPs (AQP0-AQP12) have been identified so far, and their presence has been detected in virtually all types of cells building the body [3]. Aquaglyceroporins (AQP3, AQP7, AQP9, AQP10) constitute one of the subgroups of the AQP family and facilitate the transport of water, glycerol, urea, ammonia, hydrogen peroxide (H_2_O_2_), lactate and metalloids [4]. From studies conducted mainly in laboratory animals, it is known that all four aquaglyceroporins of interest have been identified within the male reproductive system, where they exhibit cell- and tissue-specific expression patterns. AQP3 has been reported to be expressed in mouse Sertoli cells, among others, while AQP7 was identified in the seminiferous epithelium of the rat testis [5,6]. AQP9 and AQP10 were observed in the rat efferent ducts and epididymis [7].

Despite the continuous development of knowledge in the field of animal reproduction, still little is known about AQPs in the male reproductive tract in livestock, including cattle. To date, only one study has been published on AQPs in the excurrent ducts of sexually mature buffalo bulls, which tentatively identified the locations of only AQP1 and AQP9 [8]. There is no information available in the literature regarding the distribution of the remaining AQPs. It is generally known that studying the role of proteins involves, among other investigations, a detailed determination of their location, which is a necessary starting point for further studies. To date, no comprehensive studies verifying the presence and distribution of individual AQPs in the male reproductive system have been conducted either in humans or animals. The results presented below are part of a project whose main goal is to answer the question: can measuring the expression of AQPs become a modern indicator enabling a comprehensive assessment of the reproductive potential of bulls and the quality of their semen in the future? In seeking an answer to this question, the present study undertakes research aimed at determining the detailed location of aquaglyceroporins AQP3, AQP7 and AQP9 in the male reproductive system—from the testis to the vas deferens in cattle—and analyzing changes in the expression of these proteins during animal growth and development. The fourth aquaglyceroporin discovered in mammals, AQP10, was not evaluated, as its gene is a pseudogene in cattle and does not code for a functional protein [9]. In order to properly assess the distribution of individual AQPs, the present study also includes morphological analysis and identification of Sertoli and Leydig cells in all age groups of animals studied.

## 2. Results

### 2.1. Morphological Changes in the Male Reproductive Tract, Collagen Distribution and Identification of Sertoli and Leydig Cells

**Figure 1:**
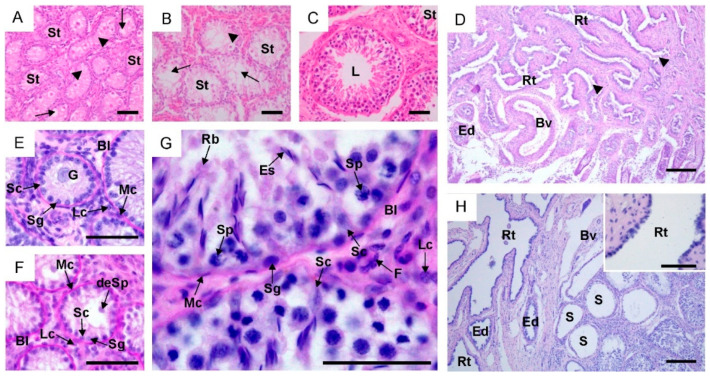
Representative light micrographs of testes, rete testis and efferent ducts in calves (**A**,**D**,**E**), young bulls (**B**,**F**) and reproductive bulls (**C**,**G**,**H**). (**A**–**C**,**E**–**G**) Testis. Bl, basal lamina; deSp, developing early spermatocytes; Es, elongating/elongated spermatids; F, Fibroblasts; G, gonocytes; L, lumen; Lc, Leydig cells; Mc, myoid cells; Rb, residual bodies; Sc, Sertoli cells; Sg, spermatogonia; Sp, spermatocytes; St, seminiferous tubules; arrowheads, mesenchymal substance; arrows, signs of tubular lumenization. (**D**,**H**) Rete testis and efferent ducts. Bv, blood vessels; Ed, efferent ducts; Rt, rete testis; S, straight tubule; arrowheads, mesenchymal substance. (**A**–**C**,**H**) Hematoxylin and eosin staining. (**D**–**G**) PAS staining. Scale bar: (**A**–**C**,**E**–**G**) = 50 μm; (**D**,**H**) = 200 μm; insert in (**H**) = 50 μm.

In all examined animals, cross-sections of the testes showed seminiferous tubules enclosed by a well-developed PAS-positive basal lamina, which was surrounded by myoid cells. In the intertubular space, Leydig cells and connective tissue cells were observed (Figure 1A–C,E–G). The diameter of seminiferous tubules significantly increased with the growth and development of animals, and it was nearly five times larger in reproductive bulls compared to calves (Supplementary Table S1). Seminiferous tubules in sexually immature animals were lined with a single layer of cells, among which Sertoli cells and spermatogonia were observed (Figure 1E,F). Large gonocytes with the pale cytoplasm were observed in the lumen of the spermatogenic tubules in calves, while smaller developing early spermatocytes were recorded in young bulls. In cross-sections of some seminiferous tubules in young bulls, migrating and differentiating gonocytes were also present. Seminiferous tubules in the first two groups were filled with a mesenchymal-like material, but its gradual atrophy and tubular lumenization were observed (Figure 1A,B). Testis of the reproductive bulls contained multilayer seminiferous epithelium with the complete process of spermatogenesis and well-defined lumen in the seminiferous tubules (Figure 1C). The seminiferous epithelium consisted of cells at different stages of spermatogenic development (spermatogonia, spermatocytes, round and elongating/elongated spermatids) located within invaginations of Sertoli cells (Figure 1G). The rete testis in calves and young bulls was lined with a single-layered cuboidal epithelium without stereocilia. In its lumen, a mesenchymal substance was observed in the youngest animals (Figure 1D). In reproductive bulls, the straight tubules were lined with a single-layered flat epithelium, the rete testis with ciliated simple cuboidal epitheliums, while the efferent ducts were lined with tall ciliated cells and short non-ciliated cells (Figure 1H).

**Figure 2:**
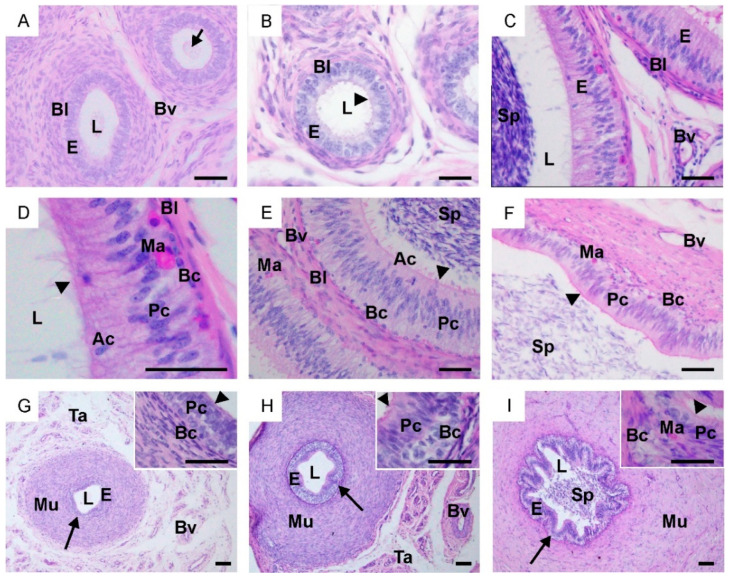
Representative light micrographs of epididymis and vas deferens in calves (**A**,**G**), young bulls (**B**,**H**) and reproductive bulls (**C**–**F**,**I**). (**A**–**D**) Caput epididymis, (**E**) Corpus epididymis, (**F**) Cauda epididymis, (**G**–**I**) Vas deferens. Ac, apical cell; Bl, basal lamina; Bc, basal cells; Bv, blood vessels; E, epithelium; L, lumen; Ma, macrophage; Mu, muscularis; Pc, principal cells; Sp, spermatozoa; Ta, tunica adventitia; arrowheads, stereocilia; arrows, lamina propria. (**A**–**I**) PAS staining. Scale bar: (**A**–**F**) = 50 μm; (**G**–**I**) = 100 μm; inserts in (**G**–**I**) = 50 μm.

Along the entire epididymal duct in calves, an undifferentiated stratified epithelium was observed, and a mesenchymal substance was found in its lumen (Figure 2A). In young bulls, the epididymis was lined with developing and differentiating epithelial cells with single stereocilia (Figure 2B). In adult bulls, along the entire epididymis, a fully developed pseudostratified columnar epithelium was observed (Figure 2C–F). Within it, basal, principal and apical cells were recognized. Basal cells were small with round or polygonal nuclei located at the basal lamina of the epithelium. Principal cells were tall with elongated nuclei, which in the caput epididymis were located near the basal lamina, while in the corpus epididymis, they were positioned at higher levels (Figure 2D,E). In the cauda epididymis, principal cells were densely arranged compared to the caput and corpus epididymis (Figure 2F). Apical cells in the caput and corpus epididymis were located closer to the lumen of the epididymal duct. These cells were not observed in the cauda epididymis. The luminal surfaces of the epithelium were covered by stereocilia, whose length decreased from the caput towards the cauda epididymis. The height of the epithelium and the tubular and luminal diameters in individual segments of the epididymis differed both within and between the groups (Supplementary Table S1). The lumen of the epididymis was filled with a large number of spermatozoa.

In both calves and young bulls, the vas deferens was lined with pseudostratified columnar epithelium consisting of basal and principal cells (Figure 2G,H). In reproductive bulls, the vas deferens was lined by a well-differentiated pseudostratified columnar epithelium. Within it, principal cells with stereocilia and basal cells were observed (Figure 2I). The epithelium of the vas deferens was surrounded by lamina propria, three layers of smooth muscle forming the muscularis, and the tunica adventitia, within which numerous blood vessels were observed. The thickness of individual layers increased with the age of the animals. Changes in the height of the epithelium, as well as an increase in its folding were observed (Supplementary Table S1; Figure 2G–I).

In the examined animals, a normal and typical distribution of collagen fibers was recorded (Supplementary Figure S1). Its content increased in all examined segments of the reproductive system along with the growth and development of the animals.

**Figure 3:**
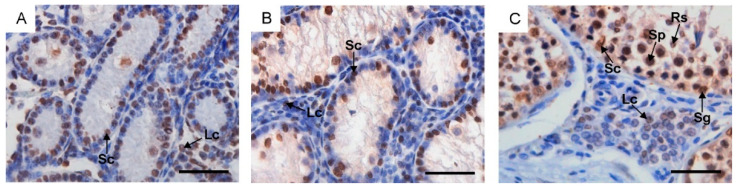
Immunohistochemical localization of GATA-4 in the testes of calves (**A**), young bulls (**B**) and reproductive bulls (**C**). Lc, Leydig cells; Rs, round spermatids; Sc, Sertoli cells; Sg, spermatogonia; Sp, spermatocytes. Scale bar: (**A**–**C**) = 50 μm.

In calves and young bulls, GATA-4 was detected in the nuclei of Sertoli and Leydig cells (Figure 3A,B). In reproductive bulls, GATA-4-positive cells also included spermatogonia, spermatocytes and round spermatids (Figure 3C).

### 2.2. Immunolocalization of Aquaglyceroporins in Bovine Male Reproductive Tract

**Figure 4:**
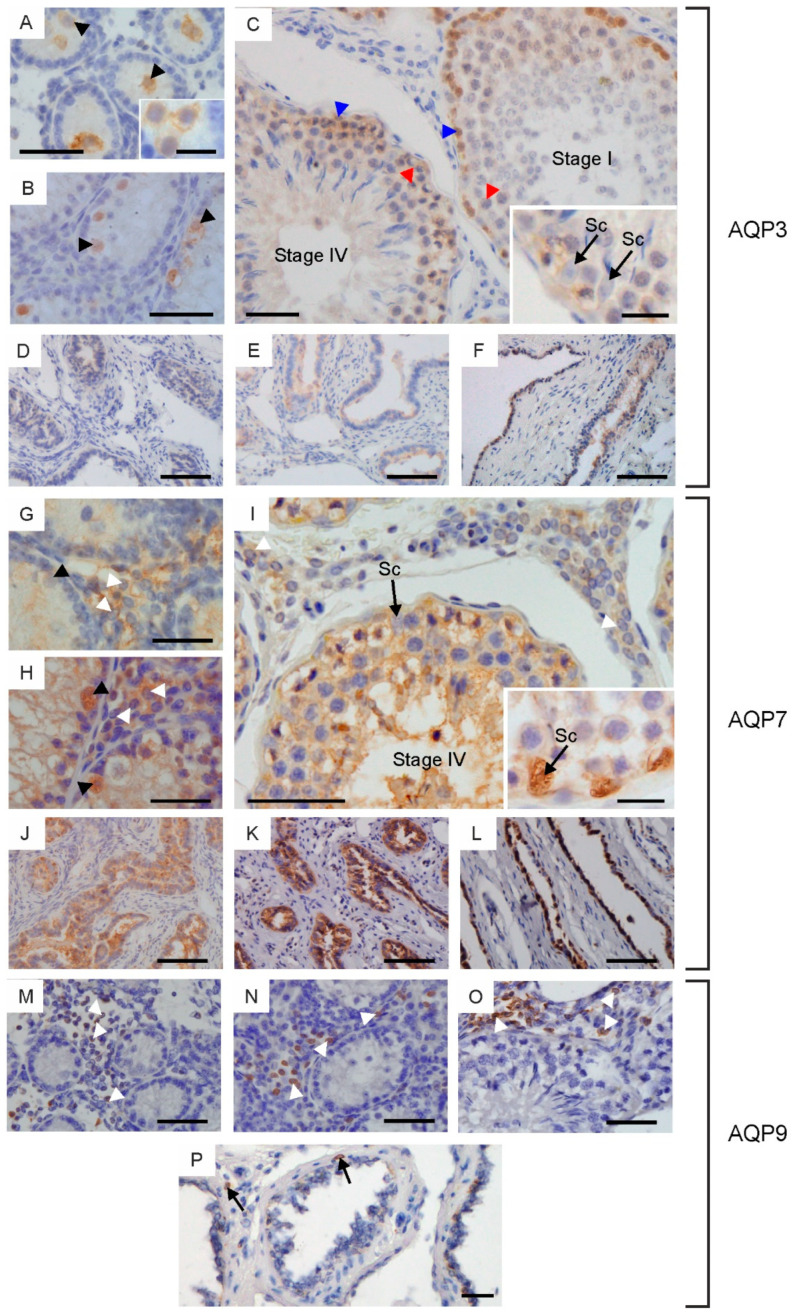
Immunohistochemical localization of AQP3 (**A**–**F**), AQP7 (**G**–**L**) and AQP9 (**M**–**P**) in the testes, rete testis and efferent ducts of calves (**A**,**D**,**G**,**J**,**M**), young bulls (**B**,**E**,**H**,**K,N**) and reproductive bulls (**C**,**F**,**I**,**L**,**O**,**P**). (**A**–**C**,**G**–**I**,**M**–**O**) Testis. Black arrowheads, gonocytes; blue arrowheads, spermatogonia; red arrowheads, spermatocytes; Sc, Sertoli cells; white arrowheads, Leydig cells. (**D**–**F**,**J**–**L**,**P**) Rete testis and efferent ducts. Arrows, stromal cells. Scale bar: (**A**–**P**), insert in (**I**) = 50 μm, inserts in (**A**,**C**) = 20 μm.

**Figure 5:**
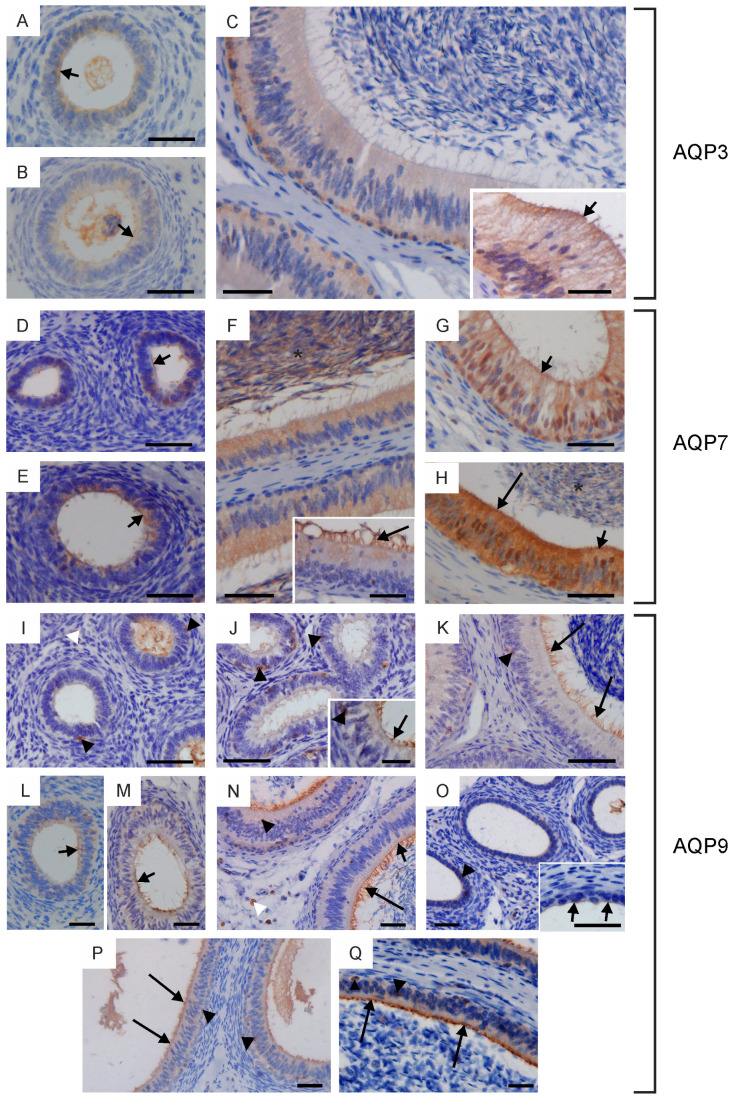
Immunohistochemical localization of AQP3 (**A**–**C**), AQP7 (**D**–**H**) and AQP9 (**I**–**Q**) in the epididymis of calves (**A**,**D**,**I**,**L,O**), young bulls (**B**,**E**,**G**,**J**,**M**,**P**) and reproductive bulls (**C**,**F**,**H**,**K**,**N**,**Q**). (**A**–**K**) Caput epididymis, (**G**,**L**–**N**) corpus epididymis, (**H**,**O**–**Q**) cauda epididymis. Arrow, stereocilia; asterisks, epididymal sperm; black arrowheads, intraepithelial cells; short arrows, apical plasma membrane of epithelial cells; white arrowheads, stromal cells. Scale bar: (**A**–**Q**), inserts in (**F**,**J**,**O**) = 50 μm, insert in (**C**) = 20 μm.

**Figure 6:**
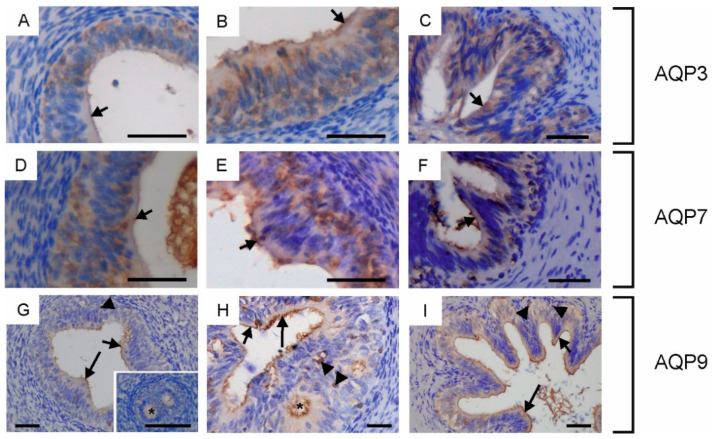
Immunohistochemical localization of AQP3 (**A**–**C**), AQP7 (**D**–**F**) and AQP9 (**G**–**I**) in the vas deferens of calves (**A**,**D**,**G**), young bulls (**B**,**E**,**H**) and reproductive bulls (**C**,**F**,**I**). (**A**–**I**) Vas deferens. Arrow, stereocilia; asterisks, intraepithelial crypts; black arrowheads, intraepithelial cells; short arrows, apical plasma membrane of epithelial cells. Scale bar: (**A**–**I**), insert in (**G**) = 50 μm.

Immunohistochemical staining revealed the presence of all three investigated AQPs in the male reproductive system in cattle (Figure 4, Figure 5 and Figure 6). The semi-quantitative assessment of the total labeling intensity of individual AQPs was carried out according to the adopted scale and is presented in Supplementary Tables S2–S5.

AQP3, AQP7 and AQP9 were expressed in the testicular tissue of the examined animals. Within the seminiferous tubules of calves and young bulls, AQP3 was visible only in gonocytes both in the cell membrane and cytoplasm (Figure 4A,B). Immunoperoxidase labeling intensity of this protein differed in cross-sections from weak to moderate. AQP7 was not detected in the seminiferous tubules of calves (Figure 4G). In some cross-sections of the testes in young bulls, AQP7 was observed in migrating and proliferating gonocytes, spermatogonia and early developing spermatocytes (Figure 4H). In both groups of sexually immature animals, AQP9 was not found within the seminiferous tubules (Figure 4M,N). In reproductive bulls, the expression of AQP3 was limited to spermatogonia and spermatocytes. This protein was visible in the cytoplasm and cell membrane, and its immunoreactivity was stronger in spermatogonia than in spermatocytes (Figure 4C). AQP3 expression in spermatogonia differed depending on the stages of the bovine seminiferous epithelium cycle (cycle assessment was performed according to Amann [10]). This protein was more abundant at stages I and II than at stages III–VIII. The presence of AQP3 in other spermatogenic and Sertoli cells was not recorded. Strong immunolabeling of AQP7 was observed at all stages of the seminiferous epithelial cycle. This protein was detected in the cytoplasm and cell membrane of all germ cells (including spermatogonia, spermatocytes and spermatids) and in residual bodies (Figure 4I). In reproductive bulls, the presence of AQP7 was observed in the cytoplasm and cell membrane of Sertoli cells (Figure 4I). In some animals, AQP7 was also visible in the nuclei of these cells (Figure 4I). In sexually mature males, no AQP9 was found in the seminiferous tubules (Figure 4O).

No AQP3 was detected in the intertubular space, while AQP7 and AQP9 were observed. In all groups of animals, AQP7 and AQP9 were found in the Leydig cells (Figure 4G–I,M–O). AQP7 was observed in the cytoplasm and cell membrane of Leydig cells, and in some cross-sections of these cells, AQP7 was also visible in the nuclei. AQP9 was mainly located in the cell membrane, less frequently in the cytoplasm of these cells. It should be noted that not all Leydig cells observed in the peritubular space were AQP7- and AQP9-positive.

In all animals studied, AQP3 and AQP7 were observed in cells lining the rete testis and efferent ducts (Figure 4D–F,J–L). AQP3 was primarily visible in the cytoplasm, less frequently in the cell membrane. On the other hand, the abundance of AQP7 was stronger in the cell membrane than in the cytoplasm. The expression of AQP3 and AQP7 in reproductive bulls within both of these structures was lower compared to younger animals. AQP9 was observed in stromal cells surrounding the rete testis and efferent ducts (Figure 4P).

AQP3, AQP7 and AQP9 were found in the entire epididymis in all animals studied. In sexually immature animals, from the caput to the cauda of the epididymis, AQP3 and AQP7 were mainly visible in the cytoplasm and less frequently in the apical cell membrane of epithelial cells (Figure 5A,B,D,E,G). Weak to moderate immunoexpression of AQP9 in the apical plasma membrane was detected in some sections of the epididymis (Figure 5L,M,O,P). In young bulls, this protein was also present in single stereocilia along the entire epididymal duct (Figure 5J). Weak to moderate expression of AQP3 was recorded in the epididymis of reproductive bulls. This protein was mainly observed in the cytoplasm and plasma membrane of the basal cells (Figure 5C). Immunoperoxidase labeling of AQP3 was also detected in the cytoplasm, plasma membrane and stereocilia of principal cells (Figure 5C). AQP7 was observed in the cytoplasm and cell membrane of principal and basal cells (Figure 5F). In some cross-sections, AQP7 was abundant in the apical part of principal cells and in their stereocilia (Figure 5F, H). A strong expression of AQP7 was recorded in the sperm present in the epididymal lumen. In young and reproductive bulls, AQP7 was observed in the nuclei of some epithelial cells of the epididymis (Figure 5G,H). In reproductive bulls, moderate to strong AQP9 staining was observed along the entire epididymis in the apical plasma membrane and stereocilia of the principal cells (Figure 5K,N,Q). In all animals studied, AQP9 was visible along the epididymis in solitary cells of epithelial and stromal tissue (Figure 5I–K,N–Q).

In the vas deferens, AQP3, AQP7 and AQP9 expression was detected in all animals at the tested ages. AQP3, AQP7 and AQP9 were visible in the cytoplasm and plasma membrane of the principal and basal cells (Figure 6A–I). In all animals studied, AQP7 was mainly observed in the apical plasma membrane of principal cells (Figure 6D–F). AQP9 was the most abundant in the apical plasma membrane and stereocilia of principal cells (Figure 6G–I). On some sections from calves and young bulls, AQP9 staining was observed at the level of the intraepithelial crypts (Figure 6G,H).

### 2.3. Immunoblotting and Densitometric Analysis of Aquaglyceroporins in the Bovine Male Reproductive System

**Figure 7:**
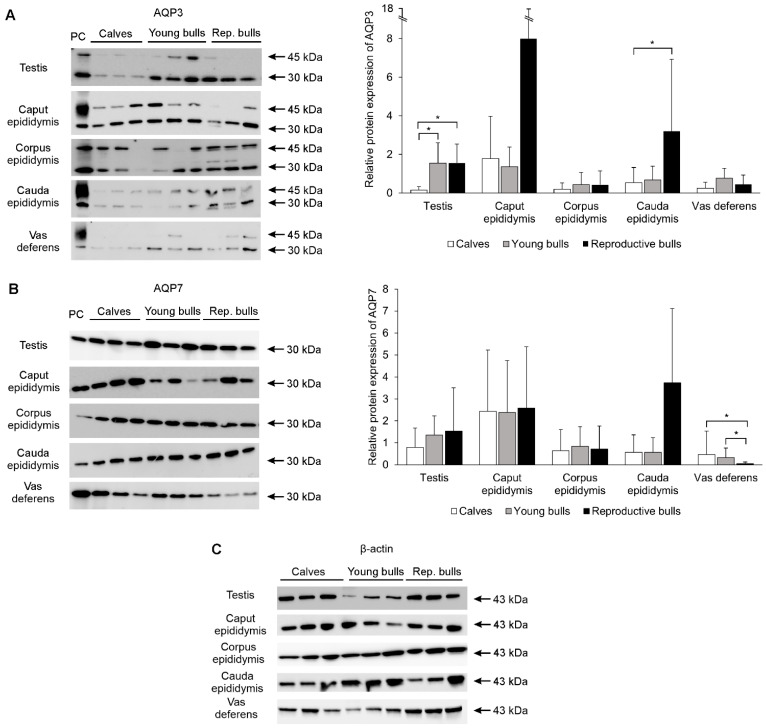
Representative Western blot images and relative protein expression of AQP3 (**A**) and AQP7 (**B**) in the bovine male reproductive tract, and blots of β-actin (**C**) used as internal standard. PC, positive control (renal medulla for AQP3 and renal cortex for AQP7). Data are presented as mean ± SD. * Significant differences in AQP expression between three age groups at the same segment of the male reproductive tract (*p* < 0.05).

Western blot analysis confirmed the presence of AQP3 and AQP7 in the reproductive system of the animals studied (Figure 7). AQP3 was detected as two distinct bands of 30 kDa and 45 kDa, corresponding to the non-glycosylated and glycosylated forms of this protein, respectively (Figure 7A). In the testes, the expression of AQP3 showed a statistically significant increase (*p* < 0.05) with age. In young and reproductive bulls, it was nearly 10-fold higher compared to calves. An increase in the relative abundance of this protein with age was also found in the caput epididymis, although it was not statistically significant (*p* > 0.05). The average optical density of AQP3 in the corpus epididymis and vas deferens in all examined animals was relatively weak and did not undergo changes between the groups. A statistically significant increase (*p* < 0.05) in the expression of AQP3 was observed in the cauda epididymis in reproductive bulls.

AQP7 was detected as a single unglycosylated band at 30 kDa in all parts of the male reproductive system (Figure 7B). The expression of AQP7 increased with age in both the testes and cauda epididymis. However, the observed increase was not statistically confirmed (*p* > 0.05). The abundance of this protein in the caput and corpus of the epididymis was relatively stable in all age groups. However, in the vas deferens, the total abundance of AQP7 was significantly lower (*p* < 0.05) in reproductive bulls compared to sexually immature animals.

## 3. Discussion

In the present study, typical and characteristic morphological changes occurring with growth and development were observed within the bovine male reproductive system. In the animals studied, these mainly included tubular lumen formation and germ cell development. In the first two groups, gonocytes that migrate to the basal membrane and differentiate into spermatogonial stem cells (SSCs) were still visible. According to Kim and coworkers [11], in cattle, the migration of gonocytes occurs when the calf is 4 weeks old, and the cells differentiate into SSCs when the animal is between 2 and 6 months of age. In young bulls, developing early spermatocytes are already visible in the lumen of the seminal tubules, indicating progressive development of germ cells.

The evolutionarily conserved zinc finger transcription factor GATA-4, required for testicular development, has been successfully used to identify Sertoli and Leydig cells [12]. In the present study, the analysis of GATA-4 expression allowed the conclusion to be drawn that in calves and young bulls, nearly half of the cells lining the spermatogenic epithelium were immature Sertoli cells, while numerous Leydig cells were observed in the intertubular space. Even though GATA-4 was observed in the nuclei of germ cells in reproductive bulls, the identification of Sertoli cells was relatively straightforward. In adult males, Sertoli cells showed lightly stained elongated nuclei, and their cytoplasm extended from the basal lamina to the lumen of the seminiferous tubule.

Consistent with previous studies [13,14], morphological changes observed at the epididymal level in the animals studied during growth included the appearance of stereocilia and the gradual differentiation of epithelial cells into basal, principal and apical cells. The above modifications were accompanied by an increase in the diameter of the tubules and lumen of the epididymis, as well as the height of the epithelium. The observed changes are typical of maturing males and prepare the epididymis for sperm transit during which spermatozoa undergo maturation, acquire progressive motility and the ability to fertilize ova [15].

The vas deferens is the least understood and studied organ of the male reproductive system [16]. However, contrary to common belief, its function appears to extend far beyond the transport of spermatozoa from the epididymis. According to Hermo and colleagues [16], in humans, the vas deferens plays a role in the viability and protection of sperm from reactive oxygen species (ROS), proteases and complement during storage. In monkeys, it is only in this final segment of the male reproductive tract where sperm achieve their full vigor and the ability to swim rapidly [17]. The observed changes and structure of the vas deferens in the animals studied are consistent with reports of other authors and indicate their progressive growth and maturation [8].

**Figure 8:**
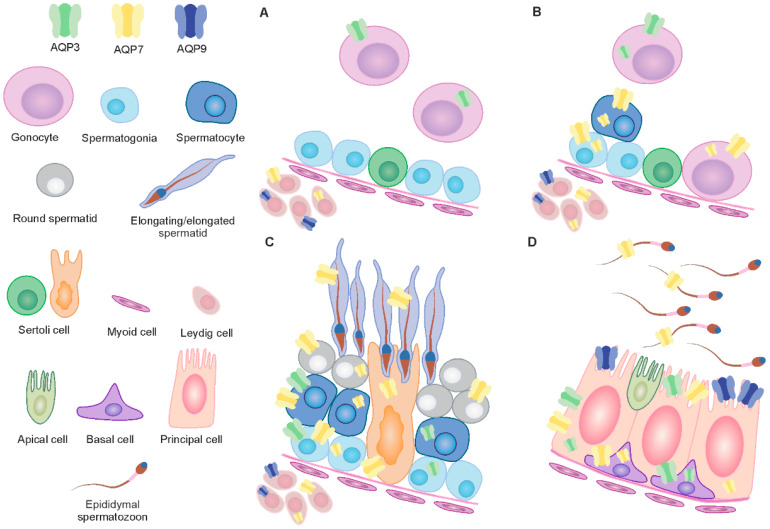
Distribution of AQP3, AQP7 and AQP9 in the bovine male gonads. The figure illustrates the localization of AQP3, AQP7 and AQP9 in the cells of the male reproductive system and their subcellular compartments (plasma membrane, cytoplasm). The different aquaglyceroporins are identified in different colors. (**A**) Testes of calves. Gonocyte—AQP3; Leydig cell—AQP7 and AQP9. (**B**) Testes of young bulls. Gonocyte—AQP3 and AQP7; Spermatogonia—AQP7; Spermatocyte—AQP7; Leydig cell—AQP7 and AQP9. (**C**) Testes of reproductive bulls. Spermatogonia—AQP3 and AQP7; Spermatocyte—AQP3 and AQP7; Round spermatid—AQP7; Elongating/elongated spermatid—AQP7; Sertoli cell—AQP7. (**D**) Epididymis of reproductive bulls. Epididymal spermatozoon—AQP7; Apical cell—not determined; Basal cell—AQP3 and AQP7; Principal cell—AQP3, AQP7 and AQP9.

The process of gonocyte formation begins in early fetal life. In cattle, gonocytes are located in the lumen of the seminiferous tubule at birth [18]. From there, they embark on the aforementioned “journey” towards the basement membrane, undergoing a series of complex changes along the way. AQP3 was found in the gonocytes of several-week-old calves (Figure 8A). However, AQP7 was also observed in young, sexually immature bulls (Figure 8B). The complex nature of gonocytes and the lack of sufficient knowledge in this area significantly hinder the interpretation of the obtained results. It seems most likely that AQP3 and AQP7 play a significant role in the migration and proliferation of gonocytes. The involvement of AQPs in the proliferation process has been pointed out by many authors [19,20,21]. According to the latest data, AQPs participate in cell proliferation through different mechanisms, including by affecting glycerol as a crucial determinant of ATP content, influencing cell cycle progression, altering the expression levels of genes relevant to this process and engaging in crosstalk with other cell membrane proteins or transcription factors. One of the factors enabling cell migration is cell volume regulation. Increased water content provides more space for actin filaments, enabling forward movement [22]. As reported by O’Flaherty and coworkers [23], extensive cell remodeling during the proliferation and migration of neonatal gonocytes is associated with the production of high levels of reactive oxygen species (ROS). According to the same authors, the main factors preventing ROS-induced cell damage are antioxidant enzymes, specifically peroxiredoxins (PRDXs). Generally, it is known that water is one of the products of H_2_O_2_ utilization. In migrating gonocytes, both AQP3 and AQP7 were observed in the cell membrane, while no other AQPs have been found in these cells to date (own research, unpublished data). Thus, it appears that both the accumulation of water, as a result of high H_2_O_2_ production, and neutralization and its efflux via AQP3 and AQP7 into the tubule lumen, which is hyperosmolar, support the process of gonocyte migration [24]. Based on the observations of Nozaki and coworkers [25], it can also be speculated that AQP3 and AQP7 observed in the cytoplasm of gonocytes promote intracellular water flow and maintain the proper volume of organelles. Undoubtedly, AQP3 and AQP7 will also enable the membrane transport of glycerol and its distribution within gonocytes. It should be noted that these aquaporins are also permeable to NH3 and urea, and AQP3 is permeable to H_2_O_2_. The results indicate that they are involved in the elimination of these compounds from gonocytes and/or enable their internal distribution. As reported by Lie et al. [26], ammonia may be an alternative nitrogen source for glutamine to promote cell proliferation. Meanwhile, the internal transport of H_2_O_2_ via AQP3 may be a component of the ROS-mediated cellular signaling cascade.

The analysis of selected AQPs in the testes of adult males has been studied by many authors. However, it should be emphasized that in the presented study, for the first time, the locations and expression of these proteins were determined during the growth and development of the animals. Within the seminiferous tubules of reproductive bulls, AQP3 expression was limited to spermatogonia and spermatocytes (Figure 8C). Previously, other authors have observed AQP3 in spermatogonia, primary spermatocytes and in the tail of elongated spermatids and spermatozoa of mouse testes [27]. A comprehensive evaluation of the localization of this protein in all the animals studied allows the conclusion to be drawn that AQP3 plays an important role primarily at the stage of growth and development of calves and the associated migration and proliferation of gonocytes. In reproductive bulls, the AQP3 expression appears to fade with successive stages of spermatogenesis. It is important to emphasize that the increase in the expression of AQP3 in the testes, as observed using WB, reflects the total amount of this protein, which undoubtedly corresponds to the increase in the body mass of animals, and consequently, testicular weight. Unlike AQP3, the expression of AQP7 in cattle increases with animal development and successively involves all germ cells. Although the abundance of this protein varies both among individuals and between different cell types, the results unequivocally indicate the significant involvement of AQP7 in the process of sperm formation and maturation. The relatively high standard deviation within the age groups of studied animals may probably result from individual variability, including hormonal status. This noteworthy observation undoubtedly requires further research in this area. As reported by many authors, AQP7 may participate in the reduction of cytoplasm during spermiogenesis, since this protein was detected in the round and elongated spermatids in most species examined to date [28,29,30,31]. In fact, up to 70% of cell volume is osmotically eliminated from the cytoplasm of round spermatids during their morphological differentiation into elongated spermatids [32,33]. In rats, the expression of AQP7 has also been reported to vary with age. Calamita et al. [34] demonstrated that the expression of this AQP increased from 45 to 90 days postnatally. According to the same authors, AQP7 may be involved in the major physiological changes in testis development and spermatogenesis.

It is well known that Sertoli cells, commonly known as “nurse cells”, play a key role in the normal development and function of the testes. These cells, among other functions, build the blood–testis barrier, participate in the formation of the proper environment in the lumen of the seminiferous tubules, provide structural and nutritional support for developing germ cells, produce and release a variety of regulatory factors and are even involved in testis immune regulation [35]. Surprisingly, in the examined animals, of all the analyzed aquaglyceroporins, only AQP7 was identified in Sertoli cells. Expression of this protein was observed in the cell membrane and cytoplasm in reproductive bulls. Based on the studies conducted so far, it is known that AQP3, AQP7 and AQP9 have been observed in rodent and human Sertoli cells [6,27,30]. According to many authors, classical AQPs located in Sertoli cells contribute to the formation of a specific tubular environment [7,36]. On the other hand, aquaglyceroporins are mainly associated with transport and the adequate supply of glycerol for developing germ cells [6]. It should be noted that the presence of AQP6 and AQP11, which also enable transmembrane glycerol transport, is not excluded in bovine Sertoli cells.

The main role of adult Leydig cells is to synthesize and release testosterone, which is known to be essential for normal male reproductive processes. The published data demonstrate that to date, AQP0, AQP1 and AQP9 have been identified in Leydig cells [7,37,38]. According to Nihei et al. [37], AQP9 is located in the cell membrane of Leydig cells surrounding the interstitial vessels, suggesting its role in the intensive water transfer between the cells and blood vessels or interstitial space. Recent data indicate that AQP9 regulates Leydig cell steroidogenesis in diabetes [39]. Hyperglycemia was shown to upregulate expression of AQP9, which in turn resulted in impaired steroidogenesis in Leydig cells through the oxidative stress pathway. In the examined animals, both AQP7 and AQP9 were identified in Leydig cells, with AQP9 mainly observed in the cell membrane and AQP7 in the cytoplasm. The results regarding the membrane localization of AQP9 confirm the suggestions of the authors cited above and reinforce the idea that this protein plays an important role in the transport of water and other solutes within the intertubular space. Intracellularly located AQP7 probably mainly regulate their distribution inside the Leydig cells.

According to common opinion, the main role of the rete testis is the simple transit of gametes from the testis to the epididymis. However, many authors rightly underscore that the function of the rete testis is much broader and includes a number of other processes, including the development of the spermatogenic system and maintenance of spermatogenesis [40,41]. The primary process occurring within the efferent ducts is the reduction of tubular fluid and associated water reabsorption [42]. To date, locations of classical AQPs have been identified and described within both of these structures, and their presence in this region, especially in the cell membrane, is relatively high [38,43,44,45]. The available data on the occurrence of aquaglyceroporins in this part of the reproductive system come, among other pieces of data, from a study by Badran and Hermo [46]. These authors observed the expression of AQP9 in the microvilli of the non-ciliated cells of the rat efferent ducts. In the animals studied, AQP3 and AQP7 were identified within the epithelium of the rete testis and efferent ducts, with the expression of AQP7 being higher, similar to the testes. The results have indicated that AQP9 is not present in cattle in this region. In the present study, it has also been found that the expression of AQP3 and AQP7 is lower in reproductive bulls. These observations allow the conclusion to be drawn that the involvement of these proteins in basic processes occurring within the rete testis and efferent ducts in sexually mature males is limited. The higher expression of AQP3 and AQP7 in the epithelium of both of these structures in calves and young bulls is difficult to explain. Perhaps, as in gonocytes, they may support the proliferation of epithelial cells, which actively occurs during development.

Spermatozoa leaving the testis enter the epididymis, where they mature, increase their motility and develop the ability to fertilize [47]. It is widely known that the proper course of these processes depends on the creation of a unique environment within the lumen of the successive segments of the epididymis. According to many authors, AQPs located in individual epididymal epithelial cells play a key role in this regard. Among the aquaglyceroporins located within the epididymis, AQP9 is currently the best characterized protein. In rats, humans, dogs, horses, buffalos and bats, this protein has been mainly observed in the stereocilia of principal cells [8,48,49,50,51]. The presence of AQP3 has been observed in the basal cells of rats and in human ciliated cells [52,53]. In contrast, AQP7 was localized in the principal cells of dogs and in the principal, basal, clear and halo cells of rats [43,52]. In the current experiment involving cattle, the presence of all three investigated aquaglyceroporins was confirmed. In sexually immature animals, AQP3 and AQP7 were mainly observed in the cytoplasm, while AQP9 was present in the apical cell membrane. The observed increase in the expression of AQP3 and AQP7 with animal growth and development indicates the progressive involvement of these proteins in the formation of the microenvironment in the lumen of the epididymis. So far, an increase in AQP9 expression in this section of the male reproductive tract was observed during development of rats and pigs [54,55]. In reproductive bulls, basal and principal cells express AQP3, AQP7 and AQP9 (Figure 8D). Both types of these cells collaborate in some way in the transport of water and/or other small solutes, removing them from the epididymal lumen throughout the epididymis [56]. Similarly to other animal species, in cattle, AQP9 is primarily located in the stereocilia of principal cells, serving as the main route for water and other molecules from the lumen to the epididymal cells. Water and other small solutes most likely exit the bovine epididymis through AQP3 and AQP7 primarily located in basal cells. It is worth noting that these aquaporins also facilitate the transport of glycerol. The results presented here confirm previous observations made by other authors that aquaglyceroporins are involved in the effective transport of glycerol from the epithelium to lumen, where it is essential for proper sperm maturation [56]. Analysis of the expression of AQP3 and AQP7 has revealed that the intensity of their immunolabeling varies both between basal and principal cells and between individuals, while AQP9 expression in stereocilia is relatively stable. Perhaps in cattle, the expression of AQP3 and AQP7, and thus the transport of water and/or other small solutes, is altered by certain factors, including hormones. Hormonal regulation of AQPs in the male reproductive system is poorly described; thus, further research in this area is highly justified. This was also indicated by the data published by Arrighi and co-workers [8] who found that in buffalo bulls, AQP9 concentrations in long principal stereocilia increased during the breeding season. It should also be noted that in the present experiment, high expression of AQP3 and AQP7 was observed in cellular compartments. Intracellular localization of these proteins reinforces the idea that they can be transported to the plasma membrane in response to hormones or kinase activation [21].

The localization of all three aquaglyceroporins in the vas deferens in the animals studied confirms earlier suggestions by other authors that this organ is not just a simple semen exit duct. The available literature lacks data on the localization of AQP3 in the vas deferens in other animal species, including humans. AQP7 was found in the dog vas deferens, where it was detected in the apical and basal regions of the epithelial cells [43]. On the other hand, AQP9 was observed in the stereocilia of the principal cells of the vas deferens in rats, bats and buffalo bulls [8,48,57]. The analysis of the distribution of AQP3, AQP7 and AQP9 suggests that, as in the epididymis, these proteins provide efficient transport of water and other small uncharged solutes from and into the lumen. The significant involvement of AQP9 in the formation of a unique microenvironment within the lumen of the vas deferens was confirmed by the previously mentioned observations of Arrighi et al. [8]. Similar to the epididymis, the expression of AQP9 in the vas deferens of buffalo bulls varied depending on the reproductive period. During the breeding period, moderate immunoreactivity of this protein was detected in the apical surface of epithelial cells, whereas no or very rare reaction was observed during the non-breeding period. Although the overall expression of AQP7 in the animals studied decreased with growth and development, this protein was the most abundant in the vas deferens among the AQPs analyzed. This indicates the leading role of AQP7 in the transport processes within this organ in cattle.

Some difficulties were encountered in optimizing the IHC method and AQP7 analysis when conducting the present research. In calves and young bulls, a clear positive signal was observed from the mesenchymal substance in the lumen of the seminiferous tubules. Cross-sections of some Leydig, Sertoli, principal and basal cells in the examined animals also showed AQP7 positive immunolabeling in their nuclei. Immunoreactivity of AQP7 was still visible despite standard measures taken to reduce background. AQPs (at the gene or protein level) were observed in the mesoderm and mesenchymal stem cells [21,58,59]. AQPs in the nuclei were also observed in the epithelial cells of the efferent ducts and the entire epididymis in rams, in the lesional and perilesional skin of vitiligo patients and in all cell types within the renal medulla in birds [45,60,61]. Some authors have suggested that selected AQPs in cancer cells in particular may affect gene expression [20]. Is AQP7 present in the mesenchymal substance and nuclei of some cells in the presented experiment, or should these signals be considered nonspecific? At this stage of the research, it is difficult to determine.

## 4. Materials and Methods

### 4.1. Animals and Tissue Collection

The experiments were conducted on male reproductive organs obtained from 31 bulls of the Polish Holstein–Friesian breed of the black-and-white variety (*Bos taurus*). Tissue samples were collected from three age groups of animals: (I) calves aged 5 to 6 weeks (*n* = 10), (II) young bulls aged 15 to 25 weeks (*n* = 10) and (III) reproductive bulls aged 2 to 6 years (*n* = 11) (Supplementary Table S6). Group I was formed using calves derived from another experimental procedure that did not affect the male reproductive system. Group II consisted of animals kept for meat, which were slaughtered at a Polish local slaughterhouse after reaching a certain body weight. The tissue samples from Group III were collected from 9 mature bulls of proven fertility used for breeding purposes and selected by the Mazovian Centre of Animal Breeding and Reproduction (Łowicz, Poland). During herd replacement, the decision was made to cull these bulls and send the animals to the slaughterhouse. Biological material from the remaining 2 animals was derived from the slaughterhouse, where reproductive bulls from a local farm were sent. The male gonads were rapidly removed directly after slaughter. After dissection, the right testes and right epididymides were weighed (Supplementary Table S7). Each region was excised and representative fragments of the testes, epididymides (caput, corpus and cauda regions) and vas deferens were cut into small uniform pieces. For histological and immunohistochemical studies, each fragment of the right reproductive organs was fixed in 10% buffered formalin, processed, embedded in paraffin block and cut into 2 μm thick sections. For Western blotting, each representative region of the left reproductive organ was frozen in liquid nitrogen and stored at −80 °C.

### 4.2. Histological and Morphometric Analysis

Deparaffinized cross-sections were stained with hematoxylin and eosin (H&E) and periodic acid-Schiff (PAS). For direct visualization and histological assessment of collagen distribution, Masson’s trichrome (MT) staining was performed. All sections were examined under a light microscope (BX43, Olympus, Hamburg, Germany) and with the use of histological scanner (Ocus^®^40, Grundium, Tampere, Finland). Scanned tissue sections were evaluated for morphometric parameters of the testes (seminiferous tubule diameter), epididymides (tubular diameter, luminal diameter, and epithelial height) and vas deferens (tubular diameter and epithelial height) using the digital ruler tool in Aperio ImageScope software (version 12.4.6.5003, Leica Biosystems, Nussloch, Germany). Tubular and luminal diameters were measured in randomly selected round or nearly round profiles representing the study regions. The epithelial heights were estimated using linear measurement between the basement and apical membrane.

### 4.3. Antibodies

All antibodies used in the present study and their dilution for immunohistochemistry and/or Western blotting are presented in Supplementary Table S8. The specificity and antigenicity of commercially available antibodies against bovine AQP3, AQP7 and β-actin were demonstrated in a previous work [62]. Homology between peptide sequences used for developing antibodies and amino acid sequences of bovine AQP9 and GATA binding protein 4 (GATA-4) was defined using the protein blast database (https://blast.ncbi.nlm.nih.gov/Blast.cgi, accessed on 1 February 2023). Depending on the type of primary antibodies, labeling of the antigen–antibody complexes was visualized with the use of secondary polyclonal goat anti-rabbit or anti-mouse horseradish peroxidase-conjugated antibodies. In the absence of commercially available blocking peptides corresponding to the used primary antibodies, traditional methods of negative controls (i.e., omission of primary antibodies) were used in immunohistochemistry and Western blot.

### 4.4. Immunohistochemistry (IHC)

Immunostaining was performed using standard immunoperoxidase procedures previously described in detail by Michałek et al. [63]. Briefly, for antigen retrieval, sections were boiled in citrate buffer at pH 6.0 (for AQP3 and AQP7) or in Tris-EGTA-buffer at pH 9.0 (for AQP9 and GATA-4). Sections were incubated overnight at 4 °C with previously validated primary antibodies against AQP3, AQP7, AQP9 and GATA-4. To eliminate background staining, testicular sections against AQP7 were incubated with specific primary antibodies for 1 h at room temperature. Samples were then incubated for 1 h at room temperature with corresponding secondary antibodies. The 3.3′-diaminobenzidine (DAB) chromogen solution (Dako, Glostrup, Denmark, cat. no. K3468) was used to visualize the immune reactions. Sections were examined using an Olympus BX43 microscope. Specificity of immunostaining was confirmed by following the above procedures in the absence of the primary antibody, which were replaced with the same amount of IgG from bovine serum (Sigma Aldrich, Darmstad, Germany, cat. No. I5506; negative controls). In the absence of a proper positive control for AQP9 in cattle, mouse liver slides were used in the experiment.

Semiquantitative evaluation of the immunostaining of AQP3, AQP7 and AQP9 was performed on tissues and the intensity of expression was scored as follows: no expression = “−”; weak = “+”; moderate = “++”; strong = “+++”.

### 4.5. Western Blot (WB)

Western blot analysis was performed as described previously by Michałek and Grabowska [62]. The membranes were incubated overnight at 4 °C with primary antibodies against AQP3 and AQP7 and the corresponding secondary antibodies (room temperature, 1 h). Signals were detected using an enhanced chemiluminescence system (ECL Prime Western Blotting Detection Reagent, GE Healthcare, Chicago, IL, USA, cat. No. RPN2232), and blot images were acquired using a ChemiDoc MP imaging system (Bio-Rad, Hercules, CA, USA). β-actin was used as an internal standard. The relative amount of proteins were determined using the Image Lab software (version 6.1.0 build 7, Bio-Rad, Hercules, CA, USA). Bovine protein extracts, i.e., renal medulla (for AQP3) and renal cortex (for AQP7), were used as positive controls. The obtained images were recorded in a digital form and modified (autoscaling was applied and a representative band was cut out) using CorelDRAW (version 21.3.0.755, Corel Corporation, Ottawa, ON, Canada). Due to the fact that the identification of Sertoli and Leydig cells was carried out to facilitate the localization of individual AQPs, no analysis of GATA-4 expression was performed using WB.

### 4.6. Statistical Analysis

All statistical analyses were conducted using Statistica (version 13.3, TIBCO Software, Palo Alto, CA, USA). The arithmetical means (X) and standard deviations (SDs) (X ± SD) were calculated. The quantitative values were evaluated using the Shapiro–Wilk normality test. To determine the significance of possible differences between the groups, the Kruskal–Wallis test with Dunn’s multiple comparison test for post hoc analysis was applied. Statistical significance was assumed at *p* < 0.05.

## 5. Conclusions

In conclusion, it should be emphasized that this study has provided for the first time a comprehensive description of the expression and distribution patterns of three aquaglyceroporins in the male reproductive system during animal growth and development. A “crude” map of the location of these proteins was created and their potential role in maturation and reproductive processes in cattle was determined. It is worth emphasizing that some observations may be useful in understanding the physiology of the male reproductive system in other animal species and humans as well. Our earlier studies on AQPs in the bovine kidney have demonstrated that both the distribution and the role of these proteins are similar to those in other mammals [62,63]. The presented results provide the necessary preliminary material for further research, which will determine, among others, the effect of various factors, including hormones, on the expression of individual AQPs. The current results, along with the extensive possibilities associated with the analysis of AQPs in the context of male reproductive potential in cattle, indicate that further studies in this area are urgently required.

## Data Availability

The dataset which supports the findings from this study is freely available at https://doi.org/10.34808/j254-s011, accessed on 25 January 2024.

## Supplementary Materials

The following supporting information can be downloaded at: https://www.mdpi.com/article/10.3390/ijms25031567/s1.
