# Supplementary material for: Cellular Distribution of Aquaporin 3, 7 and 9 in the Male Reproductive System: A Lesson from Bovine Study (Bos taurus)
Source: Int J Mol Sci. 2024 Jan 26;25(3):1567. doi: 10.3390/ijms25031567 (PMC10855163; doi:10.3390/ijms25031567)
Supplement: Supplementary file 1 [file ijms-25-01567-s001.zip › ijms-2841128-supplementary.pdf]

Supplementary Material

Cellular Distribution of Aquaporin 3, 7 and 9 in the Male Reproductive System: A Lesson from Bovine Study (*Bos taurus*)

Patrycja Oberska, Marta Grabowska, Marta Marynowska, Maciej Murawski, Dariusz Gączarzewicz, Andrzej Syczewski and Katarzyna Michałek

Table S1. Morphometric measures in the individual segments of bovine male reproductive tract in the three groups of animals.

Parameter	Age group			*P*-value
	I: Calves	II: Young bulls	III: Reproductive bulls	
**Testis**				
Sem. tub. diameter (μm)	48.1 ± 6.38[a]	75.8 ± 12.80[b]	220.5 ± 23.00[c]	0.000
**Caput epididymis**				
Tubular diameter (μm)	84.8 ± 12.43[a]	126.5 ± 21.11[b]	483.0 ± 55.83[c]	0.000
Luminal diameter (μm)	41.8 ± 10.89[a]	73.5 ± 16.95[b]	362.3 ± 47.95[c]	0.000
Epithelial height (μm)	20.6 ± 5.14[a]	27.0 ± 5.20[b]	58.1 ± 9.72[c]	0.000
**Corpus epididymis**				
Tubular diameter (μm)	98.4 ± 14.56[a]	188.1 ± 36.18[b]	435.6 ± 52.45[c]	0.000
Luminal diameter (μm)	59.2 ± 10.27[a]	100.4 ± 17.10[b]	299.2 ± 47.38[c]	0.000
Epithelial height (μm)	19.4 ± 4.22[a]	40.8 ± 12.89[b]	66.4 ± 10.29[c]	0.000
**Cauda epididymis**				
Tubular diameter (μm)	136.3 ± 55.13[a]	286.1 ± 78.90[b]	701.1 ± 155.34[c]	0.000
Luminal diameter (μm)	109.9 ± 47.47[a]	182.6 ± 70.12[b]	603.5 ± 160.64[c]	0.000
Epithelial height (μm)	10.9 ± 6.18[a]	51.3 ± 10.77[b]	37.0 ± 11.36[c]	0.000
**Vas deferens**				
Tubular diameter (μm)	228.2 ± 22.76[a]	347.0 ± 44.67[a]	568.4 ± 75.25[b]	0.007
Epithelial height (μm)	48.0 ± 8.90[a]	60.4 ± 8.28[b]	56.3 ± 8.84[b]	0.013

[a–c] Values within a row with different superscripts differ significantly at $P < 0.05$ (Kruskal-Wallis test). Data are presented as mean ± SD.

Table S2. Diagram showing staining intensity of AQP3, AQP7 and AQP9 in the bovine testis in the three groups of animals.

	AQP		
	AQP3	AQP7	AQP9
	**Testis**		
**Calves**			
Gonocytes	+/++	-	-
Spermatogonia	-	-	-
Sertoli cells	-	-	-
Leydig cells	-	+	+/++
**Young bulls**			
Gonocytes	+/++	+*	-
Spermatogonia	-	+	-
Early developing spermatocytes	-	+	-
Sertoli cells	-	-	-
Leydig cells	-	+	+/++
**Reproductive bulls**			
Spermatogonia	++	+++	-
Spermatocytes	+	+++	-
Round spermatids	-	+++	-
Elongated spermatids	-	+++	-
Residual bodies	-	+	-
Sertoli cells	-	+/++	-
Leydig cells	-	++	++

* Migrating and proliferating gonocytes. Results are expressed by a scale: - = no expression; + = weak expression; ++ = moderate expression; +++ = strong expression.

Table S3. Diagram showing staining intensity of AQP3, AQP7 and AQP9 in the bovine rete testis and efferent ducts in the three groups of animals.

	AQP		
	AQP3	AQP7	AQP9
	**Rete testis**		
**Calves**			
Epithelium	+	+++	-
**Young bulls**			
Epithelium	++	+++	-
**Reproductive bulls**			
Epithelium	+/++	++	-
	**Efferent ducts**		
**Calves**			
Epithelium	+	+++	-
**Young bulls**			
Epithelium	++	+++	-
**Reproductive bulls**			
Epithelium	+/++	++	-

Results are expressed by a scale: - = no expression; + = weak expression; ++ = moderate expression; +++ = strong expression.

Table S4. Diagram showing staining intensity of AQP3, AQP7 and AQP9 in the bovine epididymis in the three groups of animals.

	AQP		
	**AQP3**	**AQP7**	**AQP9**
	**Caput epididymis**		
**Calves**			
Epithelial cells	+/++	++	+/++
**Young bulls**			
Epithelial cells	+/++	++	++
**Reproductive bulls**			
Basal cells	++/+++	++/+++	-
Principal cells	+/++	++/+++	++/+++
Apical cells	ND	ND	ND
Epididymal sperm	-	+++	-
	**Corpus epididymis**		
**Calves**			
Epithelial cells	+	++	++
**Young bulls**			
Epithelial cells	+	++	++
**Reproductive bulls**			
Basal cells	++	++/+++	-
Principal cells	+	++/+++	++/+++
Apical cells	ND	ND	ND
Epididymal sperm	-	+++	-
	**Cauda epididymis**		
**Calves**			
Epithelial cells	+	+/++	++
**Young bulls**			
Epithelial cells	+	+/++	++
**Reproductive bulls**			
Basal cells	++	++/+++	-
Principal cells	+	++/+++	++/+++
Epididymal sperm	-	+++	-

Results are expressed by a scale: - = no expression; + = weak expression; ++ = moderate expression; +++ = strong expression. Abbreviation: ND, not determined.

Table S5. Diagram showing staining intensity of AQP3, AQP7 and AQP9 in the bovine vas deferens in the three groups of animals.

	AQP		
	**AQP3**	**AQP7**	**AQP9**
	**Vas deferens**		
**Calves**			
Basal cells	+/++	++/+++	+
Principal cells	+/++	++/+++	+/++
**Young bulls**			
Basal cells	++	++/+++	+
Principal cells	++	++/+++	++/+++
**Reproductive bulls**			
Basal cells	+/++	++	+
Principal cells	++	++	++

Results are expressed by a scale: - = no expression; + = weak expression; ++ = moderate expression; +++ = strong expression.

Table S6. Age of each individual in three groups of animals.

Animal no.	Age group		
	**I: Calves (weeks)**	**II: Young bulls (weeks)**	**III: Reproductive bulls (years)**
1	6	25	2
2	5	20	2
3	5	22	4
4	6	18	4
5	6	19	4
6	6	19	4
7	6	17	4
8	6	15	3
9	5	18	4
10	5	23	6
11	N/A	N/A	4

Abbreviation: N/A = not applicable.

Table S7. Body weight, right testicular weight and right epididymis weight in three groups of animals.

Parameter	Age group			*p*-value
	**I: Calves**	**II: Young bulls**	**III: Reproductive bulls**	
**Body weight (kg)**	63.1 ± 3.79^{a}	150.7 ± 11.26^{b}	986.1 ± 102.08^{c}	0.033
**Right testis weight (g)**	7.0 ± 1.72^{a}	24.9 ± 5.33^{b}	439.3 ± 75.29^{c}	0.033
**Right epididymis weight (g)**	1.1 ± 0.38^{a}	3.5 ± 0.75^{b}	40.4 ± 5.87^{c}	0.033

$^{a–c}$ Values within a row with different superscripts differ significantly at $P < 0.05$ (Kruskal-Wallis test). Data are presented as mean ± SD.

Table S8. Primary and secondary antibodies used for immunohistochemistry (IHC) and/or Western blot (WB).

Antibody	Dilution used for		Host	Type	Company	Cat. No.
	**IHC**	**WB**				
**Primary antibody**						
**anti-AQP3**	1:1250[1]/ 1:500[2]	**1:1000**	Rabbit	Polyclonal	Abcam	ab153694
**anti-AQP7**	1:500[1]/ 1:1000[2]	1:500	Mouse	Monoclonal	Santa Cruz Biotechnology	sc-376407
**anti-AQP9**	1:30	N/A	Rabbit	Polyclonal	Fabgennix	AQP9-901AP
**anti-GATA-4**	1:100	N/A	Rabbit	Polyclonal	Abcam	ab227512
**anti-β-actin**	N/A	1:1000	Mouse	Monoclonal	Santa Cruz Biotechnology	sc-47778
**Secondary antibody**						
**Anti-rabbit IgG (HRP)**	1:200	1:2000	Goat	Polyclonal	Dako	P0448
**Anti-mouse IgG (HRP)**	1:2000	1:2000	Goat	Polyclonal	Abcam	ab205719

[1]Antibody dilution used for testicular sections. [2]Antibody dilution used for all epididymal and vas deferens sections. Abbreviation: N/A—not applicable. Note: In order to analyze the expression and localization of AQP9 in the reproductive tract, various commercially available anti-AQP9 primary antibodies from different manufacturers, such as: Abcam (cat. no. ab84828), Santa Cruz (cat . no. sc-74409), FineTest (cat. no. FNab09925), Novus (cat. no. NBP3-12260) and Fabgennix (cat. no. AQP9-901AP) were tested. Each of the above-mentioned antibodies was verified at various dilutions both in Western blot and immunohistochemistry. In addition, mouse and bovine livers served as positive controls for AQP9. Unfortunately, none of the tested antibodies worked in the Western blot analysis.

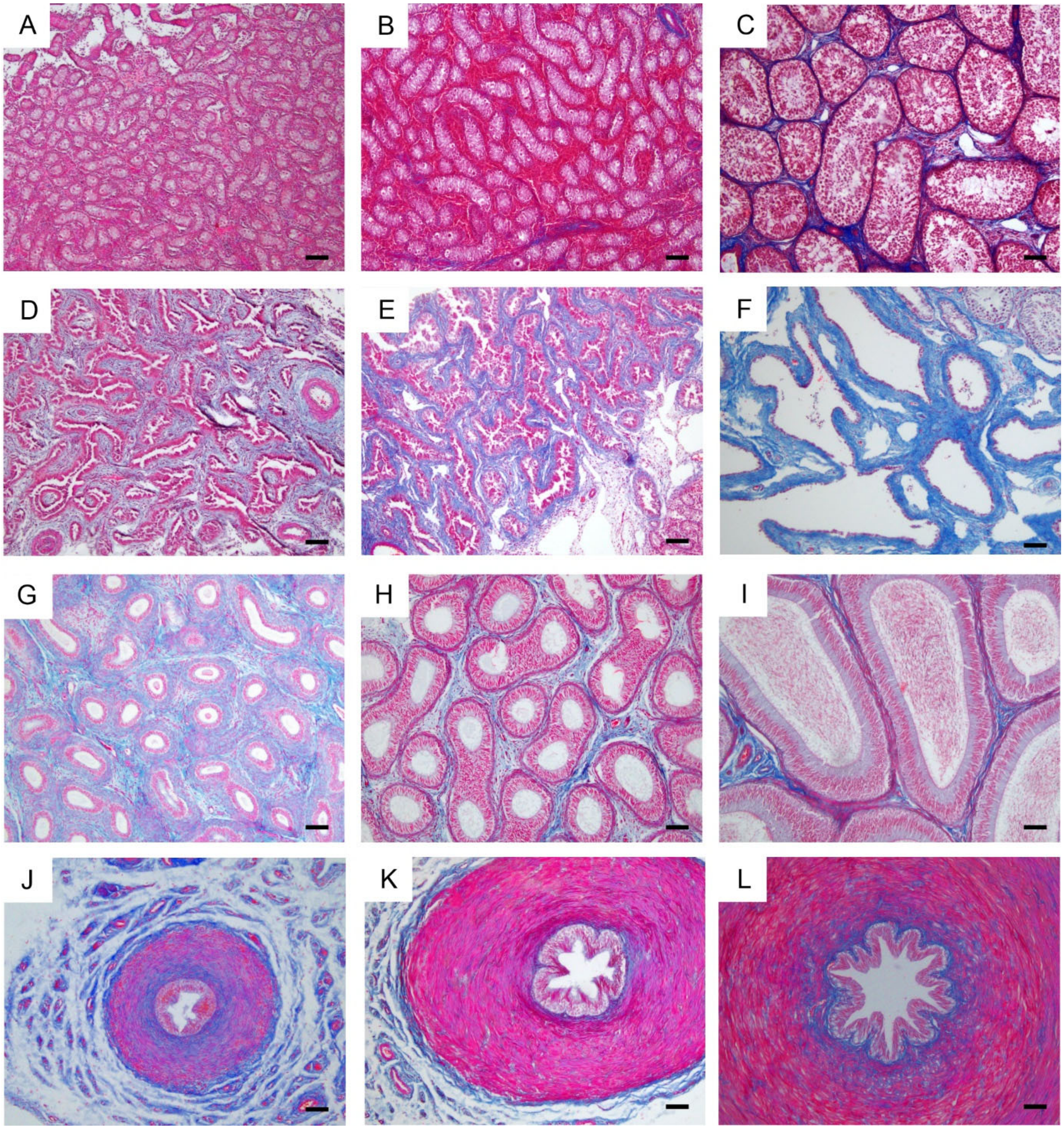

Figure S1. Representative light micrographs of bovine male organs stained with Masson's trichrome (MT) in calves (**A**,**D**,**G**,**J**), young bulls (**B**,**E**,**H**,**K**) and reproductive bulls (**C**,**F**,**I**,**L**). (**A**–**C**) Testis. (**D**–**F**) Rete testis. (**G**–**I**) Corpus epididymis. (**J**–**L**) Vas deferens. Scale bar = 50 μm.
